# Early Extracorporeal Membrane Oxygenation in COVID-19 with Bullous Lung Disease on Mechanical Ventilation: A Case Report

**DOI:** 10.5811/cpcem.2021.6.52898

**Published:** 2021-09-02

**Authors:** Jason Unold, Brandon Marshal, Tolupe Sonuyi

**Affiliations:** Detroit Receiving Hospital, Department of Emergency Medicine, Detroit, Michigan

**Keywords:** resuscitation, critical care, COVID-19, gigantic bullae, ECMO

## Abstract

**Introduction:**

Extracorporeal membrane oxygenation (ECMO) has been well described as a viable option for patients in need of temporary supplemental oxygenation when ventilator capabilities have failed to augment a patient’s condition. Less described is the potential use of ECMO for lung protection in the setting of gigantic bullae despite initially adequate oxygenation.

**Case Report:**

We describe how the early incorporation of ECMO in a patient with coronavirus disease 2019 and necrotizing pneumonia complicated by multiple large and gigantic bullae led to a favorable outcome.

**Conclusion:**

The decision to start ECMO early, despite room for ventilator oxygenation adjustments, may have helped to prevent potential, significant complications such as tension pneumothorax while on positive pressure, thus potentially optimizing the outcome in this patient.

## INTRODUCTION

Venovenous extracorporeal membrane oxygenation (VV-ECMO) is a known treatment strategy that can be used for refractory respiratory failure by running the patient’s deoxygenated blood through a membrane oxygenator before returning the blood to the area of the right atrium.^1^ This form of ECMO relies on the patient’s native cardiac function to deliver the now-oxygenated blood throughout the rest of the body. Evidence has also shown ECMO to be a useful surgical adjunct in multiple, video-assisted thoracoscopic surgeries such as treatment of a ruptured empyema, resections of gigantic bullae, difficult single-lung oxygenation surgeries, and in an iatrogenic pneumothorax in which a large bullae caused continued air leak preventing effective treatment with traditional tube thoracostomy.^2^–^5^ As demonstrated in a 2009 multicenter, randomized controlled trial, the typical indication for ECMO secondary to infections were defined as patients with severe, but potentially reversible, respiratory failure failing typical ventilator augmentations.^6^

Indications for ECMO in the setting of severe acute respiratory distress syndrome (ARDS) secondary to COVID-19 infections have yet to be defined. A review of 10 case reports and three case series failed to outline any specific usefulness of ECMO in the setting of coronavirus infections with severe ARDS sequelae.^7^

While ECMO hasn’t been shown to have specific benefits in COVID-19 infections, ECMO may be advantageous in severe but potentially reversible ARDS as well as surgical augmentation. However, ECMO as an option for lung protection in the setting of acute respiratory failure requiring mechanical ventilation with underlying gigantic pulmonary bullae has not yet been reported. It is already known that underlying bullae raise the risk for spontaneous pneumothorax.^8^ In the setting of oxygenation failure, typical treatment strategy to augment poor arterial oxygenation is administration of 100% fraction of inspired oxygen (FiO_2_) as well as increasing positive end expiratory pressure (PEEP).^9^ However, in the presence of gigantic bullae, the risk of iatrogenic pneumothorax secondary to barotrauma from positive pressure ventilation and high levels of PEEP is a significant consideration. Furthermore, unintentional placement of a chest tube within a gigantic bullae can result in iatrogenic pneumothorax, hemothorax, shock, and death.^10^

We discuss how early initiation of VV-ECMO was used to protect a patient with gigantic bullae requiring mechanical ventilation from developing an iatrogenic pneumothorax while on positive pressure.

## CASE REPORT

A young adult patient presented to our emergency department (ED) via emergency medical services (EMS) as a medical code secondary to respiratory distress and altered mentation. The unidentified male with an estimated age between 20–30 was in respiratory extremis and unable to communicate with providers. Per EMS, when they arrived at the house they found the patient in the bathtub, minimally responsive with a pulse oximetry of 60%. The patient was immediately brought to the ED. Initial Glasgow Coma Scale was six. Vital signs showed a heart rate of 161 beats per minute, blood pressure of 116/75 millimeters of mercury (mm Hg), tachypnea at 34 breaths per minute (bpm), pulse oximetry 90% on 100% FiO_2_ through bag-valve-mask, and a core temperature of 36.7° Celsius.

**Image 1:**
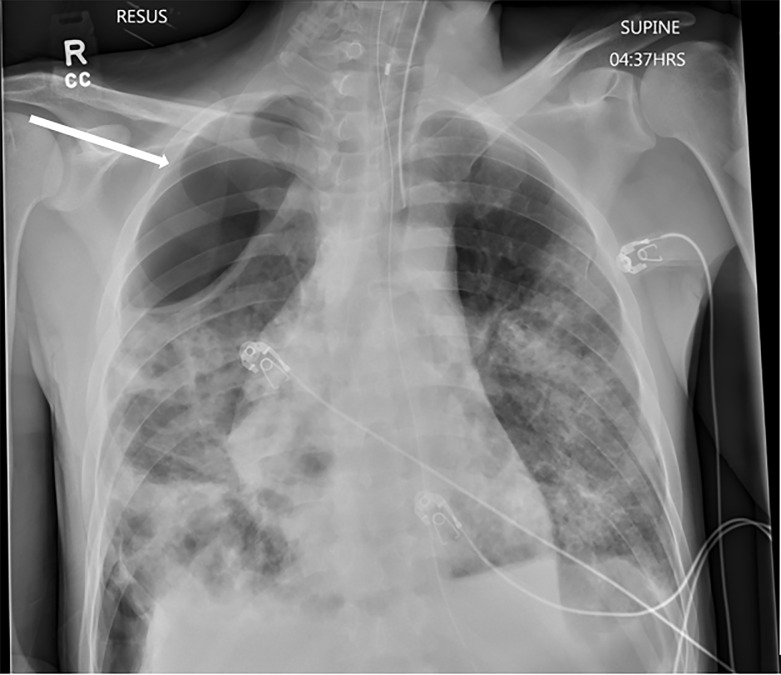
Post-intubation chest anterior-posterior radiograph demonstrating pneumonia and gigantic bulla (white arrow).

The decision was made to emergently intubate the patient and place him on mechanical ventilation. His initial ventilator settings were volume control of 450 ml (8 cubic centimeters per kilogram), rate of 16, PEEP of 5 mm Hg, and FiO_2_ of 100%. The patient was also started on broad-spectrum antibiotics and sedated with dexmedetomidine and propofol infusion. Post-intubation chest radiograph (CXR) showed bilateral interstitial infiltrates as well as a gigantic bleb in the right upper lobe (Image 1).

**Image 2:**
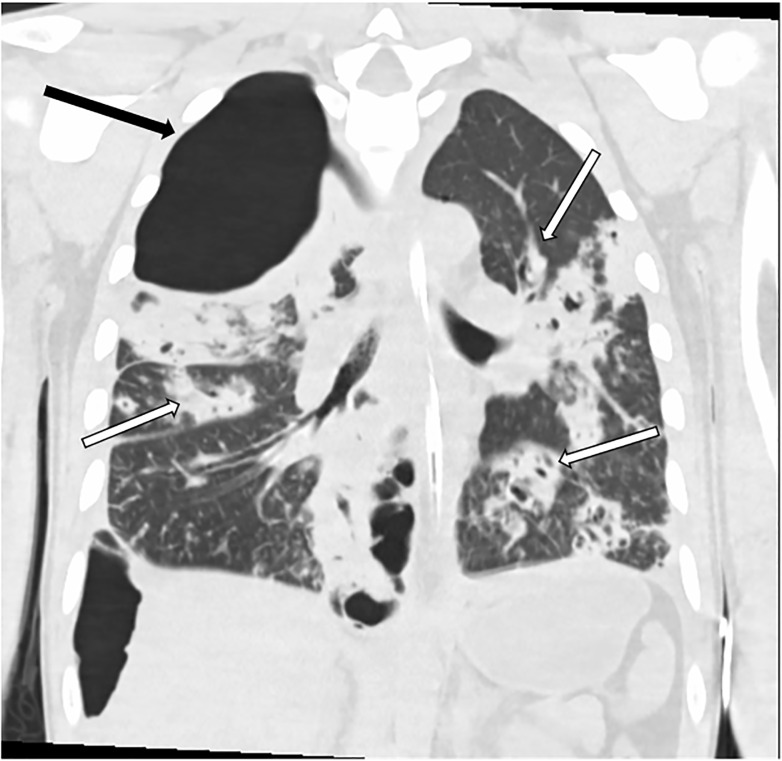
Thorax computed tomography coronal plane demonstrating right upper lobe gigantic bulla (black arrow) as well as multifocal pneumonia with bronchiectasis (multiple white arrows).

**Images 3:**
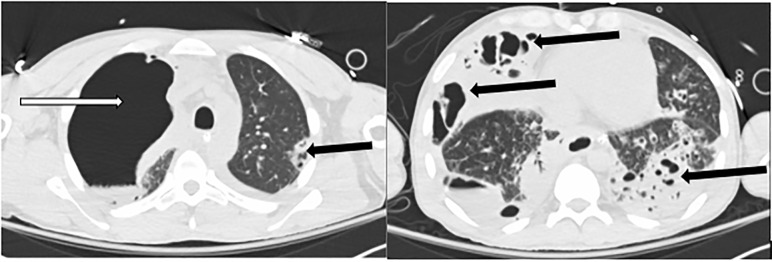
Thorax computed tomography transverse plane demonstrating giant right upper lobe bulla (white arrow) as well as multifocal pneumonia and bronchiectasis in both images at differing levels (multiple black arrows).

Thoracic computed tomography obtained while within the ED showed a necrotizing multifocal pneumonia with lower lobe predominant bronchiectasis, giant right upper lobe bulla, and large right lower lobe bulla (Images 2, 3).

Initial laboratory evaluation showed an elevated white blood cell count of 48.0 × 10^9^/liter (L) (reference range: 4.5–11.0 10^9^/L), lactic acid of 4.4 millimoles per liter (mmol/L) (0.5–2.2 mmol/L), and a positive COVID-19 screen. Initial arterial blood gas showed a pH of 7.155 (reference range: 7.35–7.45), partial pressure of carbon dioxide (PCO2) of 75.4 mm Hg (35–45 mm Hg), partial pressure of arterial oxygen (PaO2) 115.5 mm Hg (80–100 mm Hg), and bicarbonate (HCO_3_) 25.8 milliequivalents per liter (mEq/L) (reference range: 38–42 mEq/L).

A few hours into the patient’s stay, while still in the ED he developed hypotension and hypoxia to 88% on 100% FiO_2_ and PEEP of five centimeters of water (cm H_2_0). A STAT repeat CXR confirmed the absence of a pneumothorax, and bedside ultrasound showed a hyperdynamic left ventricle and flat inferior vena cava. The patient was bolused three liters of normal saline while central venous and arterial lines were placed, after which norepinephrine was initiated.

**CPC-EM Capsule**

What do we already know about this clinical entity?

*Extracorporeal membrane oxygenation (ECMO) has been shown to be advantageous in severe but potentially reversable acute respiratory distress syndrome as well as surgical augmentation.*

What makes this presentation of disease reportable?

*ECMO has not yet been reported as an option for lung protection in the setting of respiratory failure requiring mechanical ventilation with underlying gigantic pulmonary bullae.*

What is the major learning point?

*Early initiation of venovenous ECMO in the setting of gigantic bullae, may have helped to avoid an iatrogenic pneumothorax while under positive pressure ventilation.*

How might this improve emergency medicine practice?

*May lead to early identification for the involvement of ECMO equipped facilities to expedite collaboration with critical consultants.*

He then developed high peak pressures. Due to the high pressures, the ventilator settings were transitioned to pressure control with a driving pressure of 18 cm H_2_0, rate of 24 bpm, 100% FiO_2_, and a PEEP of 5 cm H_2_0. Repeat arterial blood gas showed a pH 7.19, PCO2 71.5 mm Hg, PaO2 84.7 mm Hg, and HCO_3_ of 27.2 mEq/L. The patient continued to desaturate down to 85% on 100% FiO_2_, so PEEP was increased from five to eight cmH_2_0. Because of the patient’s gigantic bullae, worsening oxygenation and hypercapnia, and concerns for inevitable iatrogenic pneumothorax, the decision was made to consult the ECMO team. Of note, at this time the patient’s arterial oxygenation was not in a critical range (84.7 mm Hg), and in a typical situation this value alone would not be a typical indication for ECMO. The ECMO team agreed that due to the complicated nature of the patient’s disease process and underlying severity of lung injury he was an appropriate candidate for VV-ECMO. The patient was taken emergently to the operating room and received a right ventricular assist device, and VV-ECMO was initiated.

Post VV-ECMO initiation, the patient was extubated in under 24 hours and transitioned to 3L nasal cannula. Sedation was weaned and the patient quickly became fully alert and was able to follow commands and communicate. On hospital day 3 his blood cultures grew positive for methicillin-resistant *Staphylococcus aureus* (MRSA). The patient received daily chest CXRs post-ECMO cannulation to tract placement and lung pathology. Post-extubation chest CXR the following day showed no new pathology; however, on the second morning post extubation he was found to have developed an asymptomatic spontaneous pneumothorax on routine chest CXR. The patient remained hemodynamically stable and did not require tube thoracostomy, potentially aiding to an outcome that would have been drastically different if he had still been on positive pressure ventilation.

The final diagnosis was established as acute hypoxic respiratory failure due to COVID-19 pneumonia with superimposed MRSA. The patient was discharged to his mother’s house with home health care and home oxygen on hospital day 29 on two liters nasal cannula.

## DISCUSSION

As demonstrated by this case, the early initiation of VV-ECMO in the setting of gigantic bullae helped to avoid an iatrogenic pneumothorax while under positive pressure ventilation, decreased the number of days on mechanical ventilation, maintained hemodynamic stability in the setting of spontaneous pneumothorax, and likely led to an overall decrease in intensive care unit stay. Very early on our patient developed signs of worsening oxygenation status despite 100% FiO_2_. Increasing this patient’s PEEP to correct for hypoxia likely would have led to positive pressure iatrogenic pneumothorax, which would have further decompensated the patient’s already poor pulmonary baseline. This hypothesis is further supported by the presence of a spontaneous pneumothorax two days post extubation. By initiating ECMO early, mechanical ventilation was discontinued prior to the development of a spontaneous pneumothorax, thus avoiding significant complications and mortality associated with a pneumothorax under positive pressure.

Our report is not without limitations. This treatment strategy requires a facility equipped with ECMO capabilities. While this may not be generalizable to all health centers, it does provide a potential strategy whereby one may begin to engage early on with nearby ECMO-capable facilities for potential transfer. Additionally, because this report was a discussion of one patient there was the possibility of unseen variables that potentially helped impact personal outcome.

## CONCLUSION

Further studies into the use of ECMO to prevent significant complications associated with gigantic bullae and positive pressure ventilation may provide useful information regarding patient care. This information may aid in the earlier identification of disease, necessity of possible transfers to ECMO-equipped facilities, expedited involvement of critical consultants, and potential for favorable patient outcomes.
